# Renal Cell Carcinoma Metastasis from Biopsy Associated Hematoma Disruption during Robotic Partial Nephrectomy

**DOI:** 10.1155/2014/975412

**Published:** 2014-12-03

**Authors:** Christopher Caputo, Ziho Lee, Andrew Harbin, Daniel Eun

**Affiliations:** Department of Urology, Temple University School of Medicine, 3401 N. Broad Street, Suite 330, Zone C, Philadelphia, PA 19140, USA

## Abstract

We describe a case in which a patient with a past medical history of ovarian cancer received a diagnostic renal biopsy for an incidentally discovered renal mass. During left robotic partial nephrectomy (RPN), a perinephric hematoma was encountered. The hematoma was not present on preoperative imaging and was likely a result of the renal biopsy. The renal cell carcinoma (RCC) and the associated hematoma were widely excised with negative surgical margins. On follow-up imaging at five months postoperatively, a recurrent renal mass at the surgical resection bed and several new nodules in the omentum were detected. During completion left robotic total nephrectomy and omental excision, intraoperative frozen sections confirmed metastatic RCC. We believe that a hematoma seeded with RCC formed as a result of the renal biopsy, and subsequent disruption of the hematoma during RPN caused contamination of RCC into the surrounding structures.

## 1. Introduction

Biopsy of small renal masses has traditionally been utilized to rule out lymphoma, abscess, or metastatic disease with a known primary tumor [1]. Although complications due to renal mass biopsy tend to be rare, hemorrhage and hematoma formation may occur near the biopsy tract. The incidence of clinically significant bleeding ranges between 0 and 1.3% [1–3]. A rare complication associated with renal mass biopsy is tumor seeding of the needle tract. This has only been described in a handful of case reports [4–7]. Herein, we describe a case in which a renal cell carcinoma (RCC) seeded hematoma formed after diagnostic renal biopsy. During left robotic partial nephrectomy (RPN), the hematoma was disrupted, evacuated, and removed with the renal mass. The patient subsequently developed RCC recurrence with diffuse metastasis to the surgical resection bed, omentum and retroperitoneum.

## 2. Case Presentation

A 61-year-old woman with a past medical history of treated stage III ovarian cancer presented with an asymptomatic new solitary left renal mass detected on surveillance imaging. Computed tomography (CT) of the abdomen and pelvis showed a 3.3 cm endophytic mass (RENAL nephrometry score 9P) [8] in the upper pole of the left kidney suggestive of a cT1a primary RCC. There was no evidence of other synchronous lesions or metastases. Given the patient's prior history of ovarian cancer, we opted for a CT-guided renal mass biopsy. Under CT guidance, a three-core biopsy was performed using an 18-gauge needle over a coaxial sheath. Pathologic examination of the mass was consistent with RCC.

**Figure 1:**
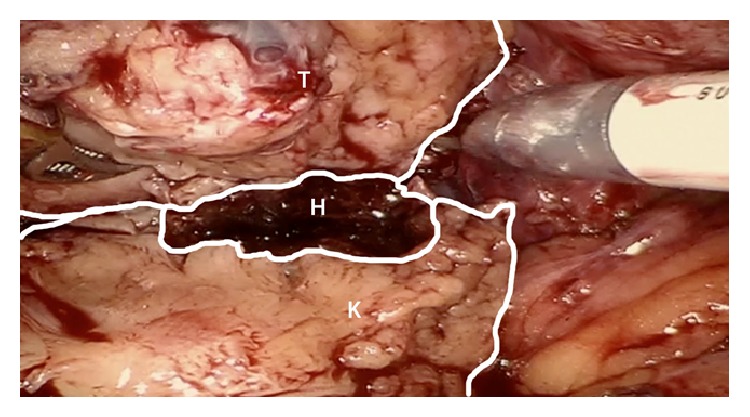
Intraoperative picture of the partial nephrectomy, demonstrating the tumor (T), hematoma (H), and kidney (K).

Given that the patient was asymptomatic and the uneventful nature of the renal mass biopsy, the lesion was not reimaged and we proceeded with transperitoneal RPN. After dissecting the renal hilum and overlying renal fat, a 12 mm laparoscopic drop-in ultrasound probe was used to evaluate the renal mass prior to resection. The mass was well visualized and there was no obvious perinephric hematoma noted. During sharp tumor resection, however, we encountered an intrarenal hematoma that was clearly outside of the tumor margin (Figure 1). The hematoma had several small areas of pale yellow coloration that appeared to be organizing hematoma. Since this was consistent with recent biopsy, we completely suctioned the hematoma and performed a wide margin resection to completely remove the tumor and hematoma. The tumor pseudocapsule was not violated during the resection. The specimen was extracted via an EndoCatch bag, and was grossly confirmed to be an intact specimen on the back table. Final pathology confirmed a 4.5 cm Fuhrman Grade 3 Papillary RCC with negative margins and an adjacent organizing hematoma.

Five months after the initial surgery, surveillance CT revealed a new 2.4 cm left renal mass, enlarged para-aortic lymph nodes, and several nodules present in the omentum. A completion robotic left total nephrectomy and omental excision was performed. Intraoperative frozen specimens of the omental nodules revealed metastatic RCC, and final pathological analysis revealed tumor characteristics consistent with those of the initial resection.

## 3. Discussion

Biopsy of a renal mass may be performed to rule out lymphoma, renal abscess, or metastatic disease in the presence of a known extra-renal malignancy. The procedure may also be performed on lesions which cannot be accurately differentiated from RCC by imaging, such as in cases of oncocytoma [3]. Most notably, renal mass biopsies have played an increasing role in determining the optimal management for small renal masses, defined as contrast-enhancing renal masses ≤4 cm in largest diameter. In particular, information regarding malignancy, histology, and grade of small renal masses may be used to support treatment decisions [2].

Traditionally, renal biopsies have been associated with low diagnostic yield, with one study reporting a 31% rate of nondiagnostic samples [9]. However, with improvements in biopsy techniques, such as the use of an 18-gauge core needle, the diagnostic yield has improved. Maturen et al. performed a single institution retrospective study on a series of 152 renal mass biopsies using a coaxial 18-gauge core needle. Of these results, only 6 (4%) were nondiagnostic, and they reported a sensitivity of 97%, a specificity of 100%, and a PPV of 100% for detection of malignant masses with a positive biopsy result [10].

Although a renal mass biopsy is generally considered to be a safe procedure, there are several known complications. The most common complication is hemorrhage from the biopsy site, which is clinically significant in <1% of biopsies [3]. Volpe et al. in 2012, performed a literature review between 1999 and 2011 examining clinically significant hemorrhage rates after biopsy. They determined that the incidence of significant hemorrhage requiring intervention such as transfusion or hospitalization ranged from 0 to 1.3% [2]. Leveridge et al. performed biopsies on 345 patients with small renal masses, and 6.4% of patients developed mild to moderate hematomas discovered on postprocedure CT or ultrasound, or postprocedure bleeding through the coaxial sheath [1].

Another potential complication of renal mass biopsy is seeding of carcinoma along the biopsy tract. Prior reports have shown that metastasis from biopsy seeding can occur along the path of the needle tract. Gibbons et al. first described this phenomenon in 1977, when they performed a posterior percutaneous biopsy on a patient with an incidental renal mass. Two years after partial nephrectomy, a 2 cm renal RCC was detected along the biopsy path [4]. In 1993, Slywotzky and Maya performed a biopsy on a patient with transitional cell carcinoma that extended into the renal parenchyma. Although radical nephrectomy was performed, the patient had recurrence of transitional cell carcinoma along the biopsy tract 8 months later [5].

In a literature review examining all published reports regarding renal mass biopsies from 1977 to 2007 by Volpe et al., only 6 case reports of tumor seeding from biopsy were found [10]. Furthermore, refinement of biopsy technique with utilization of a coaxial guide or cannula to introduce the core needle has been instituted to reduce the incidence of tumor seeding. This allows for multiple biopsy passes of core needles without contacting the surrounding structures. To our knowledge, there have been no reported cases of tumor seeding with correct usage of a guiding cannula to obtain biopsies [1–3, 10]. However, there have been documented cases of RCC seeding after improper use of the guiding cannula. Sainani et al. described RCC seeding 4 years after biopsy despite use of a coaxial sheath. The authors noted that the sheath was inserted into the mass and became a potential source for seeding the biopsy path upon its withdrawal [7].

In the present case, the patient presented with metastases several months after a technically successful partial nephrectomy. Given that local and omental recurrence 5 months after surgery would be extremely unusual, we believe that disruption of an RCC seeded hematoma during tumor resection led to tumor contamination and subsequent proliferation of tumor cells throughout the peritoneal and retroperitoneal cavity. It is unlikely that the tumor was spread along the biopsy tract during the procedure, given the distribution of the metastatic lesions, the low incidence of tract seeding in the literature [3], and the proper use of a coaxial sheath at initial biopsy. We believe that preoperative renal biopsy is an important tool for diagnosing and guiding therapy for renal masses. We highlight a rare but significant complication of the procedure that has not been previously described in the literature to the best of our knowledge.
